# The pattern of epistaxis recurrence in patients taking prophylactic acetylsalicylic acid (ASA) from a 10 year cohort

**DOI:** 10.1007/s00405-022-07666-3

**Published:** 2022-10-01

**Authors:** Petar Stanković, Stephan Hoch, Stefan Rudhart, Stefan Stojković, Thomas Wilhelm

**Affiliations:** 1 Department of Otolaryngology, Head/Neck and Facial Plastic Surgery, Sana Kliniken Leipziger Land, Borna, Germany; 2 Department of Otolaryngology, Head/Neck and Facial Plastic Surgery, Philipps-University, Marburg, Germany; 3 Department of Cardiology, Medical University, Vienna, Austria; 4 Medical Faculty, Philipps-University, Marburg, Germany

**Keywords:** Epistaxis, Aspirin, ASA, Recurrence, SCORE2

## Abstract

**Objectives:**

Epistaxis is the most common otolaryngological emergency and one-third of epistaxis patients regularly take low-dose acetylsalicylic acid (ASA) for the prevention of cardiovascular disease (CVD). The shift in contemporary guidelines identifies little benefit of ASA intake in patients who have not previously had an infarction. Existing evidence confirms ASA intake as a factor for severe epistaxis, while the evidence concerning its impact on recurrence is ambiguous. There are no available studies which justify the administration of these drugs nor are there any studies correlating the effects of these drugs to the SCORE2 CVD risk stratifying scale.

**Study design:**

A retrospective analysis of all admitted epistaxis patients in a tertiary academic hospital for the 10 year period 2011 to 2021.

**Methods:**

Patient data were analysed using the hospital information software. A recurrence was defined as an epistaxis episode requiring hospital readmittance for at least one night. Patients taking anticoagulants were excluded (*N* = 421).

**Results:**

444 patients were included: 246 were taking ASA and 198 were not (NoASA). ASA patients had more frequent recurrence in general (*p* = 0.03), more recurrences per patient (*p* = 0.002), and more changes in bleeding localisation (*p* = 0.04). Recurrence in the ASA group was associated with lower haemoglobin values (HR 0.62, *p* < 0.0001), while surgery (HR 6.83, *p* < 0.0001) was associated with recurrence in the NoASA group. ASA patients had a statistically significant (*r* 0.33, *p* = 0.032) correlation between the total number of epistaxis recurrences and SCORE2. The indication for drug intake was highly questionable in as much as 40% of ASA patients. Follow-up time was 5.27 years.

**Conclusions:**

Epistaxis patients taking prophylactic ASA are significantly more burdened by recurrence, because they have more frequent recurrences, a greater number of recurrences per patient, and more changes in bleeding localisations when compared to control patients. The drug indication is questionable in up to 40% of ASA patients, exposing them unnecessarily to recurrence.

**Level of evidence:**

4.

## Introduction

Epistaxis is one of the most common emergencies in medicine. It mostly affects the elderly and accounts for 1 in 200 general emergency department (ED) visits as well as up to one-third of otolaryngological ED visits [1, 2]. One-third of epistaxis patients are on antiplatelet therapy, most frequently acetylsalicylic acid (ASA) [3–6]. Low-dose ASA (75–150 mg) is not only traditionally widely recommended for patients who have previously suffered vascular events (secondary prophylaxis), but also for patients who have a moderately raised risk of cardiovascular disease (CVD) without previous infarction (primary prophylaxis) [7, 8] as well as the general population above the age of 55 [9, 10].

However, the latest (2019) comprehensive American Heart Association guidelines issued on the prevention of CVD question whether the use of ASA as a primary prophylaxis in patients younger than 40 and older than 70 is beneficial [11] as in this particular population group, the risk of severe haemorrhage surpasses the benefit of prophylaxis. The Canadian and European (ESC) cardiology guidelines from 2020 and 2021, respectively, concur and conclude that only a few people benefit from primary prevention [12, 13]. According to the 2021 ESC guidelines, low dose aspirin is only recommended (class II b recommendation) as primary prevention for people with either Diabetes Mellitus or a very high CVD risk [13].

ASA has been found to be associated with severe epistaxis in previous studies [3, 5, 14]. It remains unclear, however, whether ASA leads to more recurrence in epistaxis patients as the results of current studies are ambigous [5, 15]. It is also unknown what fraction of epistaxis patients taking ASA has a reasonable indication for CVD prevention, particularly in the light of current guideline changes.

The aim of our study was to comprehensively analyse epistaxis patients taking ASA, including the indication for drug intake, and compare them to control patients not taking any anticoagulant or antiplatelet drugs as well as to investigate the factors leading to recurrence. We sought to thoroughly document each recurrent in-hospital stay to investigate location changes, the intervals between recurrences and the cumulative days spent in hospital.

## Materials and methods

We performed a retrospective study analysing patient records in our hospital management software for all admitted adult epistaxis patients in the 10 year period of January 2011 to September 2021. All patients were treated at the Department of Otolaryngology, Head and Neck Surgery, Sana Kliniken Leipziger Land in Borna, Germany, a tertiary academic hospital.

We collected data on each patient’s age, gender, date and season of admission, length of in-hospital stay, intake and dosage of anticoagulants and antiplatelet drugs, the indication for antiplatelet drug(s), systolic and diastolic blood pressure (BP) and whether there was a need to lower said BP, the localisation of epistaxis (left or right side as well as anterior, posterior of diffuse), various laboratory data (haemoglobin, PTT, platelet count, INR, creatinine, grade of chronic renal insufficiency, non-HDL cholesterol), therapy, smoking status, the need for transfusion, death, and sepsis. The seasons were defined as follows: winter (December–February), spring (March–May), summer (June–August) and autumn (September–November). For each readmission epistaxis episode, the same data were collected and the interval between hospital stays for epistaxis was calculated. We noted the total number of recurrences and the cumulative length of hospital stays due to epistaxis. A recurrence of epistaxis was defined as an epistaxis episode which required hospital admittance for at least one night, and where the patient had previously been admitted to hospital for treatment of epistaxis. The Systematic Coronary Risk Estimation 2 (SCORE2) was calculated according to European guidelines for each patient [13]. The indication was marked as justified according to the most recent guidelines [11–13], secondary prophylaxis or primary prophylaxis in patients aged between 40 and 69 with SCORE2 ≥ 10. Follow-up was done until March 2022.

The main inclusion criteria was the regular intake of prophylactic ASA (100 mg/day). The control group was made up of patients taking neither anticoagulant nor antiplatelet drugs (NoASA). Accordingly, all epistaxis patients taking Vitamin-K-Antagonists (VKA), direct oral anticoagulants (DOAC) or single antiplatelet drugs other than ASA were excluded from the study. Other exclusion criteria were: septal perforation, active malignant disease, known hematopoietic diseases, and acute liver and/or kidney failure.

**Fig. 1:**
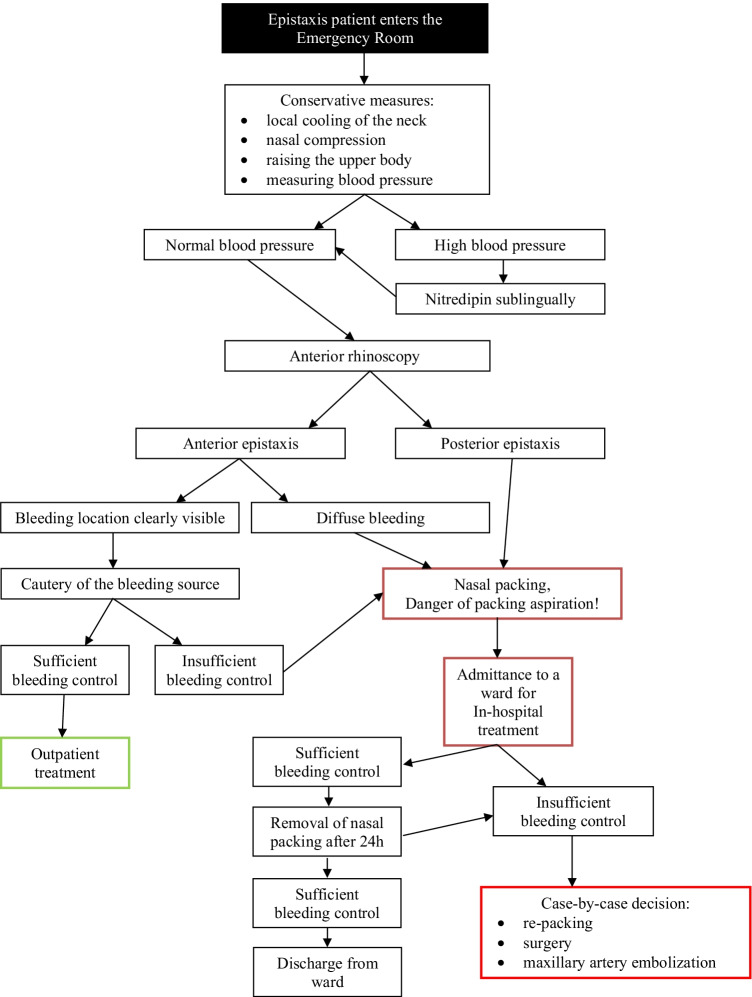
Flow-chart of the treatment of epistaxis patients (standard operating procedure—SOP)

The decision to admit an epistaxis patient for in-hospital treatment was made according to the standard operating procedure (SOP) of our clinic (Fig. 1): upon arrival in the emergency room, conservative measures were applied first, for example: local cooling of the neck, nasal compression, raising the upper body, and measuring blood pressure (BP). In patients with severely high BP, sublingual Nitrendipin was applied. Subsequently, an anterior rhinoscopy was performed to identify the bleeding localisation and in the case of an anterior epistaxis, cautery was performed. If these actions proved sufficient to stop the epistaxis and the patient was in a stable condition, he or she was discharged and the treatment was considered outpatient. If not, nasal packing was performed and the patients were admitted to a ward. This included anterior bleeders, for example, in cases where nasal packing in addition to cautery was needed for sufficient control; each patient with nasal packing must be treated in-hospital due to medico legal issues and danger of packing aspiration. In cases where these measures brought epistaxis under control, the packing was removed after 24 h and the patients were observed for a further 12–24 h before leaving the clinic. In the remaining cases re-packing, surgery or maxillary artery embolization were performed based on case-by-case decision.

Statistical analysis was performed using MedCalc^®^ Statistical Software version 20 (Ostend, Belgium). Fisher’s exact test, Pearson’s chi-squared test and Mann–Whitney *U* test was used as applicable. Univariate Cox regressions were used to perform crude and adjusted association between various predictors and recurrence. Bonferroni correction was made for multiple comparisons. The Kaplan–Meier curve was used to describe incidence of recurrence. Correlation was calculated according to Pearson’s r. The threshold for statistical significance was set at *p* < 0.05.

## Results

A total of 1039 epistaxis patients were screened for the study. After excluding 421 of those patients due to oral anticoagulant intake and another 174 due to other exclusion criteria, a total of 444 patients were included in the study, all of whom underwent stationary treatment. 246 Patients were taking prophylactic ASA (ASA-Group) and 198 patients were not taking any antiplatelet drugs (NoASA). The decision to admit the patient to the ward was always made according to the SOP of our institution. ASA patients were significantly older, with lower haemoglobin values, worse kidney function, and higher SCORE2, whereas NoASA patients displayed posterior epistaxis more often and needed BP reduction more frequently (Table 1).

**Table 1:** Comparison between epistaxis patients taking prophylactic acetylsalicylic acid (ASA) and patients not taking any antiplatelet drug (NoASA)

*N* (444)	ASA	NoASA	*p*
	246	198	
Female	99 (40.2)	90 (45.5)	0.289
Age	74.74 ± 1.48	64.14 ± 2.44	**< 0.0001**
Days in hospital	2.55 ± 0.13	2.74 ± 0.21	0.344
Recurrence	42 (17.1)	20 (10.1)	**0.034**
SCORE2 [13]	23.36 ± 1.7	15.52 ± 2.3	**< 0.00001**
Treatment			
Packing	28 (11.4)	42 (21.2)	**0.005**
Packing and cautery	201 (81.7)	134 (67.7)	**0.001**
Surgery/embolization	17 (6.9)	22 (11.1)	0.131
Localisation			
Anterior	213 (86.6)	147 (74.2)	**0.001**
Posterior	26 (10.6)	36 (18.2)	**0.027**
Diffuse	7 (2.8)	15 (7.6)	**0.028**
Systolic BP (mmHg)	147.2 ± 2.97	149.9 ± 3.77	0.217
Diastolic BP (mmHg)	83.61 ± 1.52	86.65 ± 2	**0.020**
BP lowering	21 (8.5)	36 (18.2)	**0.004**
Haemoglobin (g/dl)	12.83 ± 1.96	13.22 ± 0.26	**0.004**
Creatinine (µmol/l)	93.67 ± 4.75	88.53 ± 8.72	**0.0001**
Platelets (× 10^9^/l)	237.84 ± 10.22	242.25 ± 10.78	0.201
PTT (s)	29.4 ± 0.97	29.79 ± 1.14	0.529
INR	1.02 ± 0.02	1 ± 0.01	0.187
Chronic kidney disease			
Stage 1	38 (15.5)	64 (32.3)	
Stage 2	113 (45.9)	72 (36.4)	
Stage 3	65 (26.4)	26 (13.1)	
Stage 4	16 (6.5)	8 (4.1)	
No data	14 (5.7)	28 (14.1)	**< 0.00001**
Transfusion	4 (1.6)	2 (1)	0.696
Death	0	0	
Sepsis	0	0	

Significant *p* values are in bold

*SCORE2* Systematic Coronary Risk Estimation 2, *BP* blood pressure

**Fig. 2:**
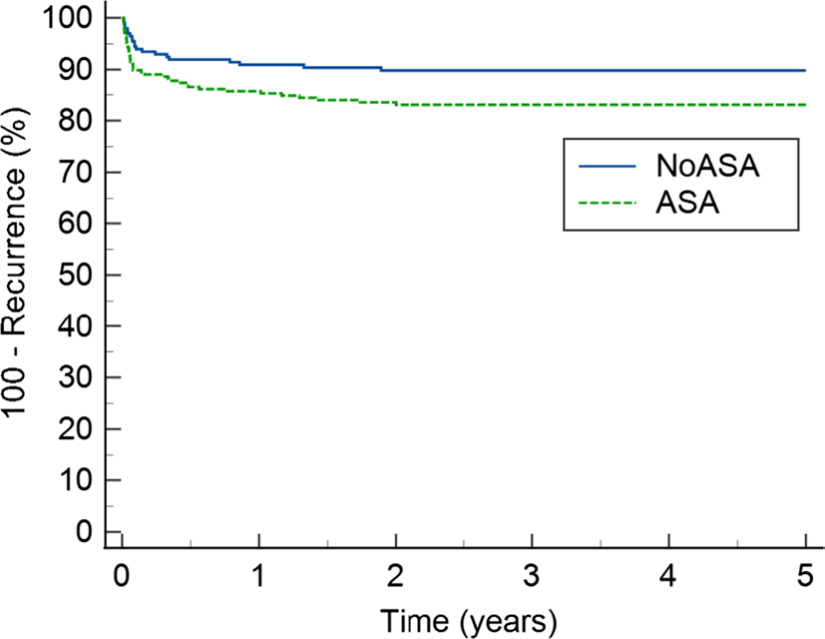
Kaplan–Meier curve of epistaxis recurrence (*p* = 0.03), *ASA* acetylsalicylic acid. Within 30 days, 59.6% of all recurrences in the ASA and 44.6% in the NoASA-Group occurred; within 180 days, 78.4% in the ASA and 80.2% in the NoASA-Group

At least one recurrence was noted in 42 patients (17.1%) in the ASA group, significantly more (*p* = 0.034) than in the NoASA group, where recurrence occurred in only 20 patients (10.1%) (Table 1, Fig. 2). According to univariate analysis, lower haemoglobin values were associated with recurrence in the ASA group. Surgery during the first admission was associated with recurrence in the NoASA group (Table 2).

**Table 2:** Univariate analysis for recurrence in epistaxis patients taking prophylactic acetylsalicylic acid (ASA) and patients not taking any antiplatelet drug (NoASA), according to Bonferroni correction, significance was set at *p* < 0.0025

	ASA 42/246			NoASA 20/198		
	HR	95% CI	*p*	HR	95% CI	*p*
Age	0.993	0.969–1.018	0.597	1.009	0.983–1.035	0.509
Female	1.122	0.609–2.067	0.713	0.963	0.399–2.324	0.933
BP lowering	1.528	0.601–3.89	0.373	0.49	0.114–2.112	0.339
Haemoglobin	0.618	0.491–0.777	**< 0.0001**	0.712	0.497–1.02	0.660
Creatinine	1.005	0.998–1.012	0.172	0.986	0.963–1.009	0.216
Platelets	1	0.996–1.004	0.833	1.004	0.998–1.009	0.188
PTT	0.944	0.769–1.16	0.584	1.075	0.913–1.267	0.386
INR	0.975	0.786–1.21	0.819	0.883	0.651–1.211	0.394
Localisation						
Anterior	0.931	0.392–2.21	0.871	0.79	0.304–2.056	0.629
Posterior	1.459	0.615–3.463	0.392	2.601	1.038–6.52	0.042
Surgery/Emb	1.582	0.565–4.433	0.383	6.829	2.786–16.74	**< 0.0001**
Transfusion	7.376	2.267–24.004	0.010			0.966
Smoking	0.348	0.084–1.438	0.145	0.609	0.141–2.626	0.506
SCORE2 [13]	0.984	0.96–1.009	0.205	1.008	0.973–1.044	0.659
Season						
Winter	1.246	0.648–2.397	0.510	0.619	0.207–1.850	0.390
Spring	1.245	0.662–2.341	0.496	3.659	1.516–8.832	0.004
Summer	0.782	0.347–1.76	0.552	0.198	0.027–1.477	0.114
Fall	0.696	0.309–1.567	0.382	0.752	0.251–2.25	0.610
DAPT	1.02	0.472–2.204	0.971			

Significant *p* values are in bold

*HR* hazard ratio, *CI* confidence interval, *SCORE2* Systematic Coronary Risk Estimation 2, *BP* blood pressure, *DAPT* dual antiplatelet therapy

There were a total of 77 recurrences in the 42 patients in the ASA group and 24 recurrences in the 20 patients in the NoASA group, resulting in 119 and 44 epistaxis in-hospital stays, respectively. The results showed that the ASA group displayed significantly more recurrence episodes per patient (*p* = 0.002), more patients exceeding 2 recurrences (*p* = 0.010), more changes of localisation on recurrence (*p* = 0.038) and more recurrences within a month after initial discharge (*p* = 0.008) (Table 3). Surgery or embolization was performed more frequently to treat recurrences in the NoASA group (*p* = 0.0009). The mean follow-up time was 5.27 ± 0.24 years (Table 3).

**Table 3:** Comparison between epistaxis patients with recurrence taking prophylactic acetylsalicylic acid (ASA) and patients not taking any antiplatelet drug (NoASA)

Recurrences	ASA	NoASA	
*N* (Patients)	42	20	
*N* (Total epistaxis episodes)	119	44	*p*
No. recurrence per patient	1.83 ± 0.47	1.2 ± 0.3	**0.002**
Cumulative days in hospital	7.83 ± 1.55	7.2 ± 1.59	0.599
Time to next recurrence (days)	155.13 ± 66.73	191.79 ± 128.79	0.952
Next recurrence < 30 days	44 (57.1)	10 (41.7)	**0.008**
> 2 Recurrences	18 (42.9)	2 (10)	**0.010**
Localisation change	15 (35.7)	2 (10)	**0.038**
SCORE2 [13]	21.14 ± 2.91	16.65 ± 5.68	**0.044**
Treatment			
Packing	14 (11.8)	7 (15.9)	0.599
Winter	8	2	
Spring	4	2	
Summer	1	1	
Fall	1	2	
Packing and cautery	100 (84)	28 (63.6)	**0.003**
Winter	33	3	
Spring	27	12	
Summer	19	3	
Fall	21	10	
Surgery/embolization	5 (4.2)	9 (20.5)	**0.0009**
Winter	2	5	
Spring	1	2	
Summer	1	0	
Fall	1	2	
Follow-up (years)	5.08 ± 0.31	5.49 ± 0.38	0.168

Significant *p* values are in bold

*SCORE2* Systematic Coronary Risk Estimation 2

**Fig. 3:**
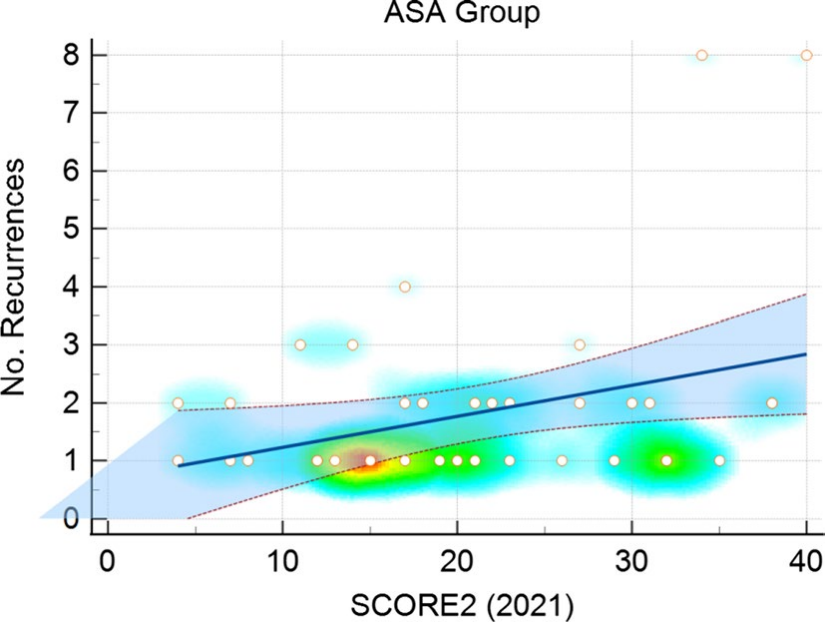
Epistaxis patients with recurrence taking prophylactic acetylsalicylic acid (ASA) (*N* = 42), showing a statistically significant (*p* = 0.03) correlation between the total number of epistaxis recurrences and SCORE2 (Systematic Coronary Risk Estimation 2), 95% confidence interval shaded blue. Spots represent individual patients with overlapping according to the heat map—bottom row from left: 4th spot 2 patients, 6th spot 5 patients, 8th spot 2 patients, 13th spot 4 patients, second row from bottom: last spot on the right 2 patients

A statistically significant correlation (*r* 0.332, 95% CI [0.031–0.578], *p* = 0.032) between the total number of epistaxis recurrences and SCORE2 was found in patients with recurrences in the ASA group (Fig. 3).

There was no appropriate indication for drug intake according to the newest guidelines in 99 out of 246 (40.2%) ASA patients. 18.2% of these patients had 1.89 ± 0.41 episodes of recurrent epistaxis, not significantly different to patients with appropriate indication, where 16.3% patients had 1.79 ± 0.31 episodes of recurrence. The patients without justifiable indication were significantly older, had a higher SCORE2, and needed surgery more often (Table 4).

**Table 4:** Comparison of epistaxis patients taking acetylsalicylic acid (ASA) according to the indication justification

	Appropriate	Inappropriate	*p*
*N* (246)	147 (59.8)	99 (40.2)	
Primary prophylaxis	58	99	
Secondary prophylaxis	89	/	
Female	53 (36.1)	46 (46.5)	0.113
Age	72.92 ± 0.9	77.43 ± 1.29	**0.0002**
< 50	1 (0.7)	4 (4)	0.161
50–69	50 (34)	16 (16.2)	**0.002**
≥ 70	96 (65.3)	79 (79.8)	**0.015**
Days in hospital	2.41 ± 0.07	2.75 ± 0.13	0.085
Posterior	12 (8.2)	14 (14.1)	0.136
Recurrence	24 (16.3)	18 (18.2)	0.732
No. of recurrence per patient	1.79 ± 0.31	1.89 ± 0.41	0.943
Surgery / Embolization	6 (4.1)	11 (11.1)	**0.041**
Transfusion	1 (0.7)	3 (3)	0.306
SCORE2 [13]	20.96 ± 1.05	27.1 ± 1.43	**0.0003**

Significant *p* values are in bold

*SCORE2* Systematic Coronary Risk Estimation 2

## Discussion

The findings of our study are that epistaxis patients taking low-dose ASA for CVD prevention are extremely burdened by recurrence. This is supported by evidence of a higher recurrence rate in the ASA group when compared to that of the control group (17.1% vs 10.1%, *p* = 0.034), the fact that more recurrences per patient occurred in the ASA group (1.83 ± 0.47 vs 1.2 ± 0.3, *p* = 0.002), and more frequent changes in bleeding localisation in the ASA group (35.7% vs 10%, *p* = 0.038). There is discrepancy in previous evidence of recurrence rates in large cohorts, some studies concurring with our findings [5, 15] and others differing [15–17]. The comparability of the groups may seem to be diminished by the older age and decreased kidney function of the ASA patients and the raised diastolic BP and increased lowering of BP in the NoASA patients. However, literature reports ambiguous results concerning the impact of old age [16–18] and BP [15, 17] on epistaxis recurrence and as a result these factors should be considered with reservation.

The recurrences were more likely to happen within 1 month of discharge in the ASA group compared to the control group (*p* = 0.008). These facts highlight pressure on the health system as each stationary treatment incurs high costs: 7000–22,000 USD in the USA [19] and 11,000 USD on average in Switzerland (with currency conversion) [20].

Regarding the dynamics of the initial recurrence episode, we found that in a follow-up period of over 5 years, 80% of first recurrence episodes in both groups occurred within 6 months of discharge. Our results are in concordance with current literature [15]. However, we are the first to report the dynamics of ASA and NoASA patients separately. These results show a steeper trend in the ASA group with 60% of all first recurrences occurring within the first month compared to 44.6% in the control group (*p* = 0.033). In addition, univariate analysis revealed low haemoglobin values as a significant factor leading to recurrence in the ASA group.

This indicates the need for intensive nasal mucosa care with ointment as well as rigorous anaemia control and post discharge treatment of these patients. Anaemia was also found to be a risk factor for recurrence, independent of ASA intake, in the study of Cohen et al. [15]. Existing evidence points out a strong relationship between lower baseline haemoglobin values and major bleeding in CVD patients [21]. Furthermore, it has been shown that low-dose ASA for primary prevention has a negative impact on the haemoglobin values of the elderly [22]. It may, therefore, be assumed that a vicious circle exists in which ASA intake in the elderly leads to lower haemoglobin values, which in turn is compounded by more frequent epistaxis recurrences.

In the light of these facts, particularly concerning the elderly with ASA intake for primary prevention, the importance of the correct indication cannot be emphasized strongly enough. We identified 157 patients (63.8%) with primary prevention as indication and 89 patients (36.2%) with secondary prevention in our cohort of 246 epistaxis patients in the ASA group. After thoroughly reviewing the patient documentation to justify primary prevention in light of the recent guideline changes, we found that 99 of the 157 patients taking ASA for primary prevention did not have a valid indication. As previously mentioned, the newest guidelines question primary prevention in patients with a low CVD risk profile who are younger than 40 and older than 70 years [11–13]. Accordingly, 40% (99/246) of patients in the ASA group in our cohort that had no justification for low dose ASA intake. This thorough analysis was unfortunately performed post-hoc and not in “real-time”, because we initially relied on the indication identified by the family doctor. Therefore, ASA was not stopped at the time of the first bleeding episode despite the fact that it was not indicated according to the most recent guidelines.

In surveys of general population, low-dose ASA is predominantly taken for primary prevention and accounts for just over 50% of all ASA intake patients, whereby 20% do not have a legitimate indication [23]. Only ¾ of primary prevention patients are in the appropriate age group of 40 to 70. This means that the potential fraction of ASA patients in the general population without a significant indication exceeds 50%.

Bearing the aforementioned treatment costs in mind, it would be interesting to calculate hospital/health system expenses for treating epistaxis in patients without a justified indication for ASA intake. These costs should be weighed against actual cardiovascular events in patients taking aspirin for primary prevention.

The patients with inappropriate indication in our cohort were significantly older, highlighting widespread ASA therapy of the elderly for inappropriate primary prevention and mirroring the recommendations of studies from the 2000s [9, 10]. The need for surgical intervention in the case of a recurrent epistaxis episode was significantly higher in this group of patients. It seems, according to our results, that a plethora of patients are exposed to unnecessary ASA intake which in turn leads to more epistaxis recurrence and more surgery for recurrence. Consequently, the indication for low-dose ASA intake needs to be verified immediately during the first inpatient treatment for epistaxis, because, as previously mentioned, up to 40% of patients do not have a reasonable indication for drug intake and most recurrences occur within 30 days.

To determine the CVD risk of patients, we estimated the systematic coronary risk using the SCORE2 scale, according to the European guidelines [13] for CVD prevention. This scale was chosen, because it is the newest, most relevant (class I recommendation [13]) and validated prediction score available. The scale range of 1–49 is determined by age, sex, smoking status, systolic BP, and non-HDL cholesterol and it stratifies patients into low to moderate, high, and very high CVD risk. This allowed the assessment of drug indication for primary prevention. On univariate analysis, SCORE2 was not a significant factor leading to recurrence in general. However, a significant relationship between the number of recurrence episodes of individual patients and SCORE2 in the ASA group was observed. Potential patients with ASA intake and SCORE2 > 20 need to be screened and drug indication thoroughly checked as this group has a high probability of 2 + epistaxis recurrences, according to our findings.

Concerning the NoASA group, we found that previous surgery was a significant risk factor for recurrent epistaxis. Bleeding points that are not easily accessible, as found in posterior epistaxis, have previously been identified as a risk factor for recurrence [16]. It can be postulated that in this group of patients, early recurrent bleeding episodes were likely the result of insufficient bleeding control in the initial treatment. This cannot be applied to the ASA group as a significant change of localisation was found in these patients. Therefore, global factors due to ASA intake must be heldaccountable.

Existing evidence states that epistaxis occurs more often in winter [18]. Our results showed that first episodes in spring led to more recurrence in the NoASA group. We defined winter according to the meteorological definition as December to February, whereas the aforementioned study used the approximate astronomical definition of January to March and registered most cases in January and March. This could explain the discrepancy. Nevertheless, it seems that seasonal variation, predominantly the cold months, plays a significant role in NoASA patients while having less impact on ASA patients.

### Limitations

Certain limitations of our study need to be disclosed. The retrospective methodology of the study is definitely a drawback as unintentional errors in data acquisition cannot be ruled out. We were able to obtain very few cholesterol values as this is not routinely done in our institution. We, therefore, approximated the SCORE2 in patients with missing values in a uniform way, using the median value. It is possible that this led to data distortion concerning this scale. We only analysed recurrence episodes requiring in-hospital treatment to determine the burden on the tertiary health system thereby excluding outpatient episodes that are very relevant to primary care.

Further noteworthy limitations are: no control group of patients taking ASA without epistaxis could be put together, data concerning bleeding in other locations as well as data about drugs interacting with ASA was not provided, and the data from excluded patients taking anticoagulants could have been used as a comparative group.

Finally, adherence to ASA therapy was not analysed in our study. It is possible that some of the ASA patients did not take ASA regularly in the days prior to admission, which may in turn have interfered with our results.

## Conclusions

The findings of our study show that epistaxis patients taking prophylactic ASA are significantly more burdened by recurrence, because they have recurrence more often, in a greater number per patient, and with more bleeding location changes compared to controls. Recurrence in ASA patients is more likely to occur in patients with lower haemoglobin values. The number of epistaxis recurrence episodes rises with a rising SCORE2. In almost half of these patients the drug indication is questionable, exposing them unnecessarily to recurrence.

## Data Availability

The authors confirm that the data supporting the findings of this study is available within the article.
